# Cell therapy of hip osteonecrosis with autologous bone marrow grafting

**DOI:** 10.4103/0019-5413.45322

**Published:** 2009

**Authors:** Philippe Hernigou, Alexandre Poignard, Sebastien Zilber, Hélène Rouard

**Affiliations:** University Paris XII, Hospital Henri Mondor 94010 Creteil, France

**Keywords:** Hip, osteonecrosis, cell therapy, bone marrow, graft

## Abstract

**Background:**

One of the reasons for bone remodeling leading to an insufficient creeping substitution after osteonecrosis in the femoral head may be the small number of progenitor cells in the proximal femur and the trochanteric region. Because of this lack of progenitor cells, treatment modalities should stimulate and guide bone remodeling to sufficient creeping substitution to preserve the integrity of the femoral head. Core decompression with bone graft is used frequently in the treatment of osteonecrosis of the femoral head. In the current series, grafting was done with autologous bone marrow obtained from the iliac crest of patients operated on for early stages of osteonecrosis of the hip before collapse with the hypothesis that before stage of subchondral collapse, increasing the number of progenitor cells in the proximal femur will stimulate bone remodeling and creeping substitution and thereby improve functional outcome.

**Materials and Methods:**

Between 1990 and 2000, 342 patients (534 hips) with avascular osteonecrosis at early stages (Stages I and II) were treated with core decompression and autologous bone marrow grafting obtained from the iliac crest of patients operated on for osteonecrosis of the hip. The percentage of hips affected by osteonecrosis in this series of 534 hips was 19% in patients taking corticosteroids, 28% in patients with excessive alcohol intake, and 31% in patients with sickle cell disease. The mean age of the patients at the time of decompression and autologous bone marrow grafting was 39 years (range: 16–61 years). The aspirated marrow was reduced in volume by concentration and injected into the femoral head after core decompression with a small trocar. To measure the number of progenitor cells transplanted, the fibroblast colony forming unit was used as an indicator of the stroma cell activity.

**Results:**

Patients were followed up from 8 to 18 years. The outcome was determined by the changes in the Harris hip score, progression in radiographic stages, change in volume determined by digitizing area of the necrosis on the different cuts obtained on MRI, and by the need for hip replacement. Total hip replacement was necessary in 94 hips (evolution to collapse) among the 534 hips operated before collapse (Stages I and II). Sixty-nine hips with stage I osteonecrosis of the femoral head at the time of surgery demonstrated total resolution of osteonecrosis based on preoperative and postoperative MRI studies; these hips did not show any changes on plain radiographs. Before treatment, these 69 osteonecrosis had only a marginal band like pattern as abnormal signal and a volume less than 20 cubic centimeters. The intralesional area had kept a normal signal as regards the signal of the femoral head outside the osteonecrosis area. For the 371 other hips without collapse at the most recent follow up (average 12 years), the mean preoperative volume of the osteonecrosis was 26 cm^3^ (minimum 12, maximum 30 cm^3^). The mean volume of the abnormal signal measured on MRI at the most recent follow up (mean 12 years) was 12 cm^3^. The abnormal signal persisting as a sequelae was seen on T1 images as an intralesional area of low intensity signal with a disappearance of the marginal band like pattern.

**Conclusion:**

According to our experience, best indication for the procedure is symptomatic hips with osteonecrosis without collapse. In some patients who had Steinberg stage III osteonecrosis (subchondral lucency, no collapse) successful outcomes (no further surgery) has been obtained between 5 to 10 years. Therefore in selected patients, even more advanced disease can be considered for core decompression. Patients who had the greater number of progenitor cells transplanted in their hips had better outcomes.

## INTRODUCTION

Osteonecrosis (aseptic avascular bone necrosis) is a relatively frequent disorder. When not consequent to trauma, it can be associated with steroid usage, alcoholism, storage disorders, vaso-occlusive episodes such as fat emboli or sickle cell disease, and some autoimmune disease, but a considerable fraction of osteonecrosis are idiopathic. The common features are apparently a decrease of osteogenic progenitors, increase of bone cell death and altered intramedullar vascularity, which are potentially all causally related.

Core decompression as treatment was described by Arlet and Ficat^1^ as early as 1964. The aim of the technique is to improve repair in the osteonecrotic segment at least at earlier stages before mechanical failure of the femoral head has occurred. Reconstruction repair has been observed after core decompression, but usually this repair is incomplete. New vessels and bone cells from a theoretical point of view arrive in the dead bone along the channel of the core decompression. In the adult, hematopoietic red marrow is normally absent in the femoral head, but red marrow containing stem cells persist in the proximal shaft of the femur. One of the reason for bone remodeling leading to an insufficient creeping substitution after osteonecrosis in the femoral head may be the small number of progenitor cells in the proximal extremity of the femur with osteonecrosis of the femoral head. While fundamental research and clinical studies have shown that dead bone may be repaired by living bone, the reparative osteogenic potential is slight in osteonecrosis: the number of bone progenitor cells in the uninvolved part of the femoral head and in the trochanteric region is less than in healthy subjects.^2^,^3^ Because this lack of progenitor cells, treatments modalities should stimulate and guide bone remodeling to sufficient creeping substitution to preserve the integrity of the femoral head. At this time, using progenitor cells or growth factors may be one of the solutions. The treatment of osteonecrosis with bone marrow autografts is based upon the view, now commonly held, that the osteogenic cells derive from a stem cell in the bone marrow stroma. When red bone marrow is transplanted,^4^,^5^ the graft will contain osteogenic precursors, which will repopulate the osteonecrotic bone. In the early stages of the disease, the femoral head is still round. By definition, the necrotic zone will be acellular, at least as far as osteocytes and bone marrow cells are concerned. However, before collapse, the bone framework is still intact; in particular, it will have retained its strength, even though the cell population in the upper end of the femur is abnormally small. This is why it was thought that conventional core decompression should be supplemented by an autograft of cells harvested by bone marrow aspiration from the ipsilateral iliac crest.

## MATERIALS AND METHODS

Between 1990 and 2000, 342 patients (534 hips) with avascular osteonecrosis at early stages (Stages I and II) were treated with decompression and autologous bone marrow grafting.

In the current series, grafting was done with autologous bone marrow obtained from the iliac crest of patients operated on for osteonecrosis of the hip. From 1990 to 2000, 1359 hips with osteonecrosis were operated on; therefore, the 534 hips in the current series represent only 39% of the hips with osteonecrosis treated at the current authors' institution.

Bone marrow aspiration: Bone marrow harvesting is most conveniently done by two surgeons working simultaneously on one anterior iliac crest each. Under sterile conditions and general anesthesia, a 2-mm skin incision is made at the level of the anterior iliac crest. The needle for bone marrow aspiration is then pushed

A beveled metal trocar of 6–8 cm length and a bore of 1.5 mm is pushed deep by hand about 6 cm into the cancellous bone of the iliac crest so that the tip lies between the inner and outer tables. A 10 mL syringe that has been flushed with heparin is used to aspirate the marrow. If no marrow is obtained, the needle should be reoriented. Once the needle has been inserted to the desired depth, the tip is swept around a full circle in 45° steps, with the bevel pointing in different directions at each step. Bone marrow is withdrawn at each of these points. Once this 360° aspiration has been performed at one site, the needle is brought out and reinserted at a different site, where the 360° sweep in 45° steps is repeated. This procedure is continued until a sufficient quantity of bone marrow has been harvested. The cell content of the marrow thus obtained will be greater^6^ if the marrow has been aspirated in small (2 mL) fractions, since, under these conditions, the proportion of contaminating peripheral blood will be less. The same percutaneous tract may be used for multiple punctures of the iliac crest. All the marrow aspirated is discharged into a plastic collection bag containing ACD (acid citrate dextrose) anticoagulant solution. It is then filtered, to remove fat aggregates and clots. Bone marrow harvesting is most conveniently done by two operators, each working on one iliac crest. An assistant places the aspirated material in the collection bag and flushes the syringes with heparin.

Bone marrow concentration: The aspirated material needs to be reduced in volume in order to increase its stem cell content. This is done by removing some of the red blood cells (the non-nucleated cells) and the plasma, in such a way as to retain only the nucleated cells, i.e., the mononuclear stem cells as well as the monocytes, the lymphocytes, and some granulocytes. This involves a considerable amount of bone marrow handling. Although the technique as such is suitable for large volumes of marrow, great care must be taken to ensure that everything is done with full sterile precautions so as to obtain a product that is safe for re-injection. Two techniques are available: bone marrow may be harvested, concentrated, and re-injected under the same anesthetic, in which case concentration must be performed sufficiently speedily so as not to exceed a time of 30 min, or the harvested material may be frozen and re-injected at a later stage; in this case, there is no constraint on the time taken to concentrate the marrow.

For re-injection under the same anesthetic, a Cobe 2991 blood cell washer was used. With this technique, the bone marrow is centrifuged for 5 min at 400 g (g = gravity). Leucapheresis is performed during 40 to 50 s at a collection rate of 100 mL/min. This centrifuging technique yields a “concentrated myeloid suspension” of 50 mL stem cells, from a 300 mL volume of aspirated bone marrow; the stem cell concentrate is placed in a syringe for reinjection.

Bone marrow grafting in the femoral head: Patients were placed on a table with two image intensifiers with a C arm. The decompression was done with a percutaneous approach using a three mm diameter trephine (trocar of Mazabraud, Collin, France). The instrument (trocar of Mazabraud, Collin, France) is introduced through the greater trochanter, as in conventional core decompression. Its position in the femoral head and in the necrotic segment is monitored with fluoroscopy. Since, at the time of treatment, the plain radiographs will show little if any evidence of necrosis, the preoperative MRI scans should be used together with the image intensifier views, to determine the site of the lesion. The tip of the trocar is positioned by using a brightness amplifier. Unrecognized joint penetration may occur because radiograph beam projects an equatorial dimension of the femoral head on to the film. The incidence does not enable determination of whether the extremity of the trephine is situated inside or outside of the femur head if the end of the implant lies slightly outside of the subchondral zone, since, in the projection, it may show within the femur head. At this time, the femur head should be rotated in the acetabulum to obtain various radiographic incidences of the head or, conversely, the screen intensifier may be rotated around the femur head to examine its contours and prevent “blind” spots. It should, however, be borne in mind that the rotation of the screen intensifier in a single plane reduces the “blind” zones but does not completely eradicate them.

Although the bore of the trocar is small compared with the trephines normally used for core decompression, femoral head and trochanteric region pressure measurements have shown that even a small hole relieved the intraosseous pressure. During the bone marrow injection, the pressure in the femoral head was found to rise, but a normal pressure pattern was restored once the injection was finished, exactly as in intraosseous pressure measurements. In our patients, no complications were observed during anesthesia; in particular, there was no reduction in oxygen saturation and no change in the pulse rate or blood pressure.

Patients were allowed weightbearing using crutches for 10 days after surgery and were full weightbearing without crutches thereafter. Patients were followed up from 8 to 18 years. The outcome was determined by the changes in the Harris hip score, by progression in radiographic stages, by change in volume determined by digitizing area of the necrosis on the different cuts obtained on MRI and by the need for hip replacement.

## RESULTS

Patients were followed up from 8 to 18 years with a mean follow-up of 13 years. Thirty- two patients have a follow-up of 18 years. The mean age of the patients at the time of decompression and autologous bone marrow grafting was 39 years (range: 16–61 years). No complications were recorded during the anesthesia. No pulmonary embolism, thrombophlebitis, or intertrochanteric fracture was observed. The outcome was determined by the changes in the Harris hip score,^7^ by progression in radiographic stages according to Steinberg *et al.* classification^8^ and by the need for hip replacement. Total hip replacement was necessary in 94 hips (evolution to collapse) among the 534 hips operated before collapse (Stages I and II). Patients who did not require total hip arthroplasty (n = 440) had a mean Harris hip score of 70 points preoperatively and 88 points postoperatively. Total hip arthroplasty was necessary in 94 hips. The average total number of colony-forming units obtained from bone marrow aspiration was estimated to be 27 × 10^3^ cells after culture of samples *in vitro*. After concentration (cell recovery: 84.3%), the average total number of colony-forming units injected by hip was estimated to be 24 × 10^3^ cells. In each group of patients with different etiologic factors, the number of transplanted progenitor cells was different and had an influence on the outcome of the hips. Hips that received a low number of transplanted cells had a more significant risk of failure at the latest follow up than hips that received a high number of transplanted cells. This may explain in part the influence of the etiologic factors on the outcome of the hips. The number of progenitor cell counts (in terms of fibroblast colony-forming units) was lower (3 times less) in the patients who had osteonecrosis attributable to corticosteroid therapy, alcohol abuse, or organ transplantation than in the patients who had osteonecrosis attributable to sickle cell disease or other causes. This may be in relation with the toxicity of steroids and of alcohol on the progenitor cells.

**Figure 1:**
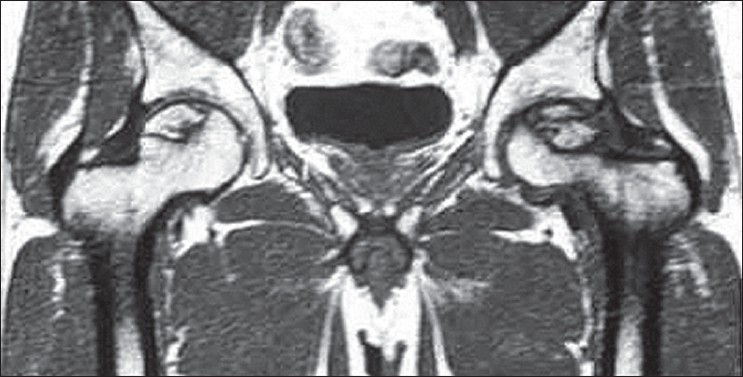
Preoperative MRI (T1 weighted images) of both hips showing bilateral avascular necrosis

**Figure 2:**
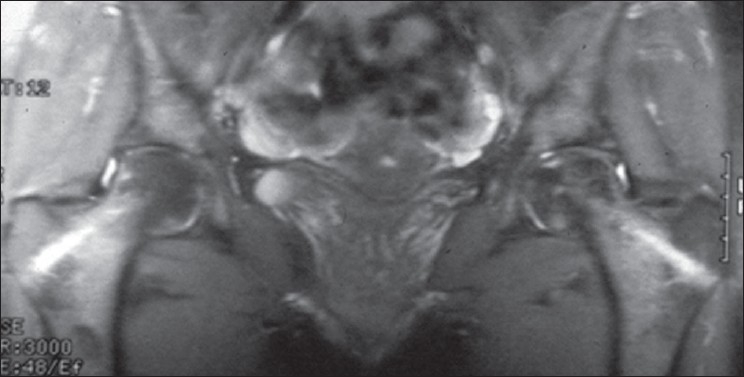
Immediate postoperative MRI (T2 weighted images) after core decompression and autogenous bone marrow grafting showing the channel and changes of signal in the superior part of the femur

**Figure 3:**
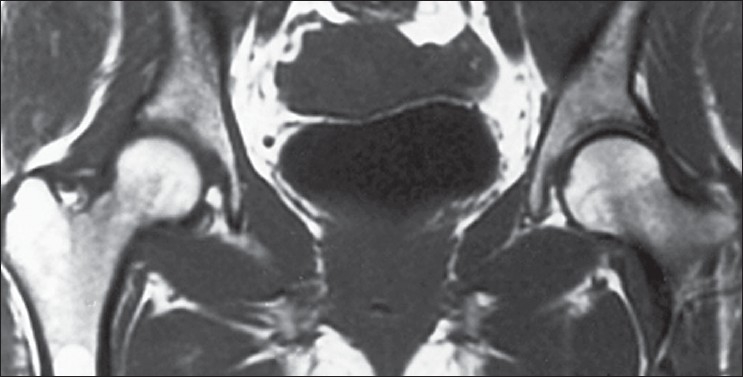
The signal appears normal in both hips after 3 years follow up showing good vascularisation

Sixty-nine hips demonstrated total resolution of osteonecrosis based on preoperative [Figures 1–3] and postoperative MRI studies; these hips did not show any changes on plain radiographs: All these 69 hips had stage I osteonecrosis of the femoral head at the time of treatment with autologous bone marrow grafting. Before treatment, these 69 osteonecrosis had only a marginal band like pattern as abnormal signal and a volume less than 20 cm^3^. The intralesional area had kept a normal signal as regards the signal of the femoral head outside the osteonecrosis area. For the 371 other hips without collapse at the most recent follow up (average 12 years), the mean preoperative volume of the osteonecrosis was 26 cm^3^ (minimum 12, maximum 30 cm^3^). The mean volume of the abnormal signal measured on MRI at the most recent follow up (mean 12 years) was 12 cm^3^. The abnormal signal persisting as a sequelae was on T1 images an intralesional area of low intensity signal with a disappearance of the marginal band like pattern. Patients who had the greater number of progenitor cells transplanted in their hips had better outcomes.

## DISCUSSION

During normal fetal and postnatal development, bone marrow is fully engaged in hematopoiesis. The process of conversion from the red to the yellow fat-storing bone marrow begins early and continues until 25 years of age, when the adult pattern of distribution is reached.

The distribution of hematopoietic marrow in the proximal femur is related to various factors. MRI studies have indicated that the conversion of red to fatty marrow may occur in patients with avascular necrosis at the upper end of the femur. In patients with osteonecrosis following corticosteroid therapy, abnormalities have been also demonstrated in the bone marrow of the iliac crest, with a decrease in the stem-cell pool.^9^ Steroids induce *in vitro* the fat-storing phenotype in bone-marrow progenitor cells.^10^ Since bone marrow fat-storing cells and osteoblasts share a common pool of stem cells, and steroids stimulate the differentiation of marrow stem cells into fat-storing cells, a decrease in the stem-cell pool may result in low number of mesenchymal progenitor cells. Their number may become insufficient to provide enough osteoblasts to meet the needs of bone remodeling, and this can result in bone necrosis. The lack of bone cell progenitors is thus one of the causes of osteonecrosis and hence a potential target for therapies. Simultaneously, intramedullary vascularity is altered and this may be a predisposing factor for osteonecrosis, since bone marrow blood supply and bone remodeling are linked. The lack of osteogenic cells could also influence two different events in the pathogenesis of osteonecrosis: first, the occurrence of osteonecrosis itself and then the bone repair which should occur after osteonecrosis. Taken together, these data have elicited the proposal for autologous bone marrow cell grafting for treatment of osteonecrosis in 1990.^2^

Autologous bone marrow grafting is also able to obtain a long-lasting repair in hip osteonecrosis. Reconstruction repair has been already observed after core decompression, but this repair is usually incomplete. One of the reasons may be the small number of progenitor cells in the femoral head of patients with osteonecrosis. Although fundamental research and clinical studies have shown that dead bone may be repaired by the living one, the reparative osteogenic potential is low in osteonecrosis: the number of bone progenitor cells in the uninvolved part of the femoral head and in the trochanteric region is lower than in healthy subjects.^3^ Whether this decrease in progenitor cells is a cause or a consequence of the necrosis is not known and this will require additional studies. However, because of this lack of progenitor cells, treatment modalities should stimulate and guide bone remodeling by increasing the availability of bone progenitor cells in order to preserve the integrity of the femoral head.

Finally, the decreased vascularity of the femoral head is one of the hallmarks of osteonecrosis. Bone-marrow derived mononuclear cells are able to elicit formation of new blood vessels by the presence of endothelial cell progenitors or hemangioblasts in this cell fraction.^11^–^15^ This may be due both to supply of progenitor cells and to angiogenic cytokines produced by bone marrow cells. Endothelia progenitors can actively engage in vasculogenesis in tissue devoid of vessels, and in neoangiogenesis from the pre-existing capillaries. Besides the generation of new capillaries, the growing endothelia enhance mobilization and growth of mesenchymal progenitors through angiopoietin1-Tie2 pathway, which generate pericytes and vascular mural cells required for new vessel growth and stabilization. A broad capacity of differentiation of perivascular mesenchymal cells has been shown and participation of perivascular mesenchymal progenitors in repair of adjacent tissues has been described both in experimental models and humans. On its turn, local ischemia that activates the cytokines signaling and mobilization of circulating progenitor may supply permanent stimuli for blood vessel repair and supply of new cells for bone regeneration. These mechanisms may explain the long-lasting effect of cell therapy in femoral head necrosis. The kinetics of neoangiogenesis in cell-mediated repair of bone necrosis is unknown, and this deserves extensive studies. Autologous bone marrow transplantation was proposed in 1990 for the treatment of osteonecrosis by Hernigou.^2^ Using progenitor cells, growth factors, or gene therapies inducing angiogenesis may be one of the solutions for this chronic and progressive debilitating hip lesion.

The number of CFU-Fs in bone marrow was determined by assessing the number of nucleated cells and the prevalence of CFU-Fs among them. The number of CFU-Fs which were placed in the graft was determined by the concentration in the original bone marrow aspirate and by centrifugation. The prevalence of connective tissue progenitors in bone marrow in the iliac crests of patients was approximately one per 30000 nucleated cells. According to the mean nuclear cell count per mL (18 × 10^6^ cells), the bone marrow harvested by aspiration from the iliac crest contained on average approximately 600 progenitors per mL in our series. After preparation of the bone marrow this concentration was increased to approximately 2500 progenitors per mL. Each site of osteonecrosis received a mean of 30 mL of bone marrow graft. The influence of the number of progenitors on the treatment of osteonecrosis has been evaluated with MRI. Each femoral head received a mean of 30 mL of bone marrow graft. According to the mean nuclear cell count per mL (29 × 10^6^ cells), the bone marrow harvested by aspiration from the iliac crest contained, on average, approximately 1160 progenitors per mL, which increased the bone marrow concentration to approximately 4900 progenitors per mL. Comparing the MRI taken before and after grafting, the volume of the repair was estimated to be 7 cm^3^ at one year, 13 cm^3^ at two years and 16 cm^3^ after four years.

One of the authors (PhH) has clinical experience with aspiration of bone marrow in more than 1000 patients.^16^–^21^ No complications were encountered. Recent publications have confirmed the efficiency of this procedure.^21^,^22^,^23^

## CONCLUSION

According to our experience, best indications are symptomatic hips with osteonecrosis without collapse. In some patients who had Steinberg stage III (subchondral Lucency, no collapse) successful outcomes (no further surgery) has been obtained between 5 to 10 years. Therefore in selected patients, even more advanced disease can be considered for core decompression.

Another indication may be sometimes discussed: it is the treatment of the asymptomatic contralateral hip during the same anesthesia. However, at this time, the literature on asymptomatic osteonecrosis is not very clear on the degree of risk for progression of asymptomatic early-stage disease.
